# Predicting Infectious Diseases: A Bibliometric Review on Africa

**DOI:** 10.3390/ijerph19031893

**Published:** 2022-02-08

**Authors:** Paulina Phoobane, Muthoni Masinde, Tafadzwanashe Mabhaudhi

**Affiliations:** 1 Department of Information Technology, Central University of Technology, Free State, Private Bag X200539, Bloemfontein 9300, South Africa; emasinde@cut.ac.za (M.M.); Mabhaudhi@ukzn.ac.za (T.M.); 2 Centre for Transformative Agricultural and Food Systems, School of Agricultural, Earth and Environmental Sciences, University of KwaZulu-Natal, Private Bag X01, Pietermaritzburg 3201, South Africa; 3 International Water Management Institute (IWMI-GH), West Africa Office, PMB CT 112 Cantonments, Accra GA015, Ghana

**Keywords:** infectious diseases, prediction, bibliometric review, Africa, research hotspots

## Abstract

Africa has a long history of novel and re-emerging infectious disease outbreaks. This reality has attracted the attention of researchers interested in the general research theme of predicting infectious diseases. However, a knowledge mapping analysis of literature to reveal the research trends, gaps, and hotspots in predicting Africa’s infectious diseases using bibliometric tools has not been conducted. A bibliometric analysis of 247 published papers on predicting infectious diseases in Africa, published in the Web of Science core collection databases, is presented in this study. The results indicate that the severe outbreaks of infectious diseases in Africa have increased scientific publications during the past decade. The results also reveal that African researchers are highly underrepresented in these publications and that the United States of America (USA) is the most productive and collaborative country. The relevant hotspots in this research field include malaria, models, classification, associations, COVID-19, and cost-effectiveness. Furthermore, weather-based prediction using meteorological factors is an emerging theme, and very few studies have used the fourth industrial revolution (4IR) technologies. Therefore, there is a need to explore 4IR predicting tools such as machine learning and consider integrated approaches that are pivotal to developing robust prediction systems for infectious diseases, especially in Africa. This review paper provides a useful resource for researchers, practitioners, and research funding agencies interested in the research theme—the prediction of infectious diseases in Africa—by capturing the current research hotspots and trends.

## 1. Introduction

Infectious diseases remain the major cause of morbidity and mortality globally [1]. They are also a major hindrance to development, especially in developing and underdeveloped countries [1,2]. Infectious diseases such as malaria, diarrhea, and tuberculosis are among the top ten causes of death in developing countries [3]. The severe acute respiratory syndrome coronavirus 2 (later named coronavirus disease 2019, COVID-19 or coronavirus) has recently been identified as one of the world’s deadliest pandemics [4,5]. The World Health Organization (WHO) report on 4 January 2022 reveals that the COVID-19 pandemic has infected up to 281,808,270 people and claimed 5,411,759 lives globally [6]. In Africa, the confirmed cases were 7,164,485 cases with 155,675 deaths [6]. Even though Africa is among the continents with the lowest number of COVID-19 confirmed cases, Africa has a long history of novel and re-emerging infectious disease outbreaks such as malaria and Ebola [7,8]. The impact of infectious disease outbreaks in Africa has been severe because of the economic, political, climatic and environmental impacts, and the many more challenges the continent already faces [9]. Infectious diseases account for over 227 million lives lost and produce an annual productivity loss of over US$800 billion yearly on a global scale [10].

Several approaches, ranging from traditional methods to mathematical models and more recently machine learning-based approaches, have been used to predict infectious diseases [11,12,13,14,15,16,17,18]. For instance, Sharma et al. [19] and Masinde [15] developed machine learning classification models to predict malaria outbreaks in India and Africa. Guo et al. [12] developed an artificial intelligence-based model to predict dengue outbreaks in China. Artificial intelligence and machine learning algorithms such as deep learning, decision trees, and artificial neuron networks have shown very high performance of up to 99% accuracy in classification and computation performance [15,20]. Moreover, other researchers have developed weather-based prediction systems for infectious diseases such as malaria and dengue using meteorological factors such as temperature and rain [15,21,22,23].

On the other hand, Macherera and Chimbari [16] developed an early warning system for malaria prediction based on Zimbabwe’s indigenous knowledge. Many of these methods assist in diagnosis, classification, risk forecasting, and predicting future disease outbreaks [24]. Infectious disease prevention and control measures such as vaccinations can be planned for and implemented before disease outbreak seasons. The effective prediction of infectious diseases can enhance a community’s resilience towards infectious disease [25,26,27] outbreaks and the resulting shocks.

Due to an increased frequency of infectious disease outbreaks and the associated impacts, many research projects have been conducted to predict Africa’s infectious diseases, resulting in an ample number of scientific publications [15,21,22,28,29,30]. A considerable number of systematic reviews provide a comprehensive analysis of the literature on predicting infectious diseases in Africa [25,26,27,31]. However, only a few of these focus on the bibliometric analysis aspect [32]. Moreover, some research studies only focus on specific infectious diseases instead of infectious diseases as a whole, and they do not necessarily focus on the prediction aspect [33,34]. Bibliometric analysis is a quantitative method based on statistics and mathematics techniques that studies the aspects of publications, dissemination, and use [35]. Bibliometric analysis can identify the main research areas within a scientific field, indicate the research development, hot spot topics, and research gaps in a specific research area [36]. Moreover, unlike in systematic review, bibliometric analysis quantitatively gives insight into the collaboration patterns of authors, institutions, and countries and their performance in a specific research domain [37]. To the best knowledge of the authors of this paper, a bibliometric analysis of the prediction of infectious diseases in the context of Africa has not been conducted.

This study aims to assess the advancement in research on the prediction of infectious diseases in Africa. In doing so, two rigorous bibliometric software, VOSviewer version 1.6. 15 and R studio version 3.4.3, are used. To achieve the overall objective, two sub-objectives were investigated: (1) to analyze the relationship among infectious disease occurrence and the progression of publication and (2) to identify research trends, topics, and gaps related to Africa. This was driven by the hypothesis that; “*providing a bird’s-eye view and understanding of the available knowledge in the research of the prediction of infectious diseases in Africa would contribute to combating infectious diseases and paving the way for future research opportunities this field*”.

## 2. Materials and Methods

The data sources used in this bibliometric analysis were the published papers retrieved from the Web of Science core collection (WoS). To improve the search, we used the advanced search option to combine diverse topics related to predicting infectious disease outbreaks. The search was restricted to predicting infectious diseases, with Africa being the geographical boundary of this study. There were no restrictions set for language, the document type, or period of review study (January 1996 to 1 October 2021). Building a valid search query is one of the challenges in any bibliometric study. While the aim is to retrieve a maximum number of papers, irrelevant papers should be excluded [38]. In the infectious diseases research field, a wide range of search strings could be used. In this paper, the search strings used to identify publications were infectious diseases that are common in Africa, ‘infectious disease’, ‘prediction’, ‘forecast’, ‘machine learning’, ‘artificial intelligence’ and ‘Africa’. These search strings were used in combination using ‘OR’ and ‘AND’ Boolean operators. Consequently, the search topic was set as follows to search for publications on the prediction of infectious diseases in Africa and those that used machine learning or artificial intelligence:

TS = (‘infectious disease *’ or ‘COVID-19’ or ‘malaria’ or ‘Ebola’ or ‘plague’ or ‘Measles’ or ‘Yellow Fever virus’ or ‘monkeypox’ or ‘Zika Virus’) AND TS = (‘forecast *’ or ‘predict *’ or ‘machine learning’ or ‘artificial intelligence’) AND TS = (‘Africa’) 

**Figure 1:**
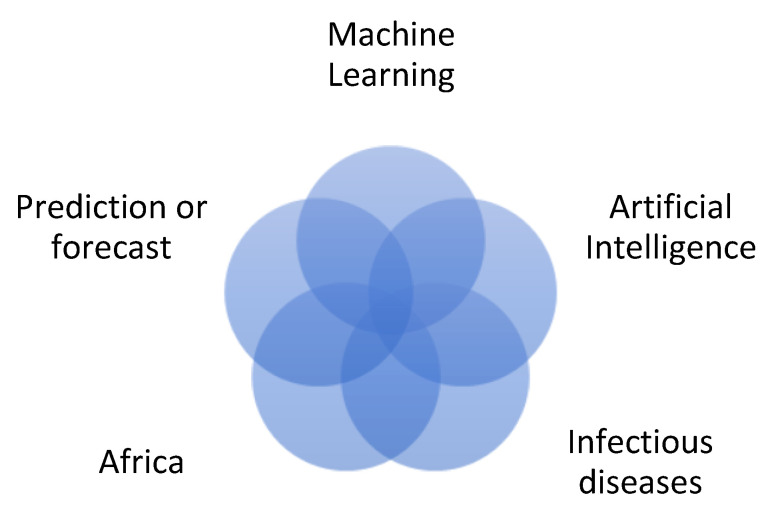
The intersection of the four literature review topic categories.

The query was done on the topic fields which entail paper titles, abstracts, keywords, and indexing fields. Figure 1 below illustrates the intersection of the four literature review topic categories considered in this review. The retrieved data was the bibliographic metadata about the publications and their citing publications. This comprised of information about authors, document, content, citation, and funding. While the information about authors included names, affiliations, address, open researcher, and contributor ID (ORCID), the document information specified document type, publication date, journal title, issue and volume among many. On the other hand, content information included paper title, abstract, and keywords, and citation information reflected reference lists and number of citations. Funding information indicated funding agency and grant number.

**Figure 2:**
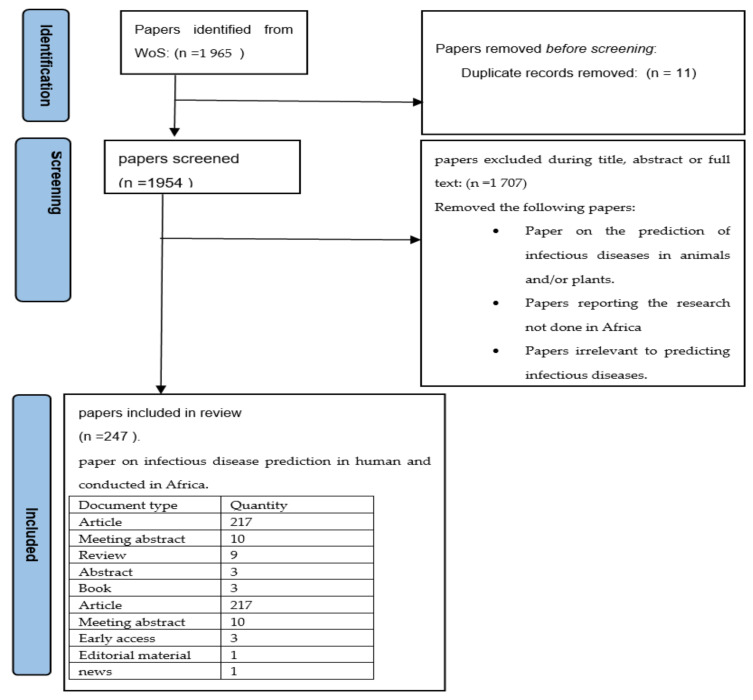
Process of identifying papers for inclusion.

The search resulted in a total of 1965 papers. However, we screened the retrieved documents at the title and abstract or full text to improve the quality of the data and, consequently, some documents that did not meet the inclusion criteria were excluded. The inclusion criterion was that the paper should be regarding infectious disease prediction in humans and conducted in Africa. The excluded documents included publications on the prediction of infectious diseases in animals and/or plants, the documents on the research not done in Africa, and duplicates or documents irrelevant to predicting infectious diseases. The filtering process resulted in 247 papers that met the inclusion criteria. The retrieved data included different document types, such as articles, reviews, books, and conference papers. Figure 2 below depicts the process of identifying papers for inclusion and the types of papers considered in the prediction of infectious diseases in Africa.

VOSviewer version 1.6. 15 and bibliometric R package were used to analyze the retrieved data and generate network maps. VOSviewer is a bibliometric software used to create maps from network data and visualize and explore those maps [39]. On the other hand, the bibliometric R package is statistical computing software [40]. The following analyses were conducted to get an overall structure of infectious disease prediction: co-citation analysis, keywords co-occurrence analysis, collaborations analysis, and thematic analysis.

This paper proceeds as follows: Section 3 evaluates publication trends and identifies emerging themes around the infectious disease outbreaks research. The fourth section focuses on the assessment of gaps and possible research opportunities. The last section, Section 5, concludes the paper by detailing its main contribution and pointing out its further research.

## 3. Results

### 3.1. Predicting Infectious Disease Outbreak Research Trends

**Figure 3:**
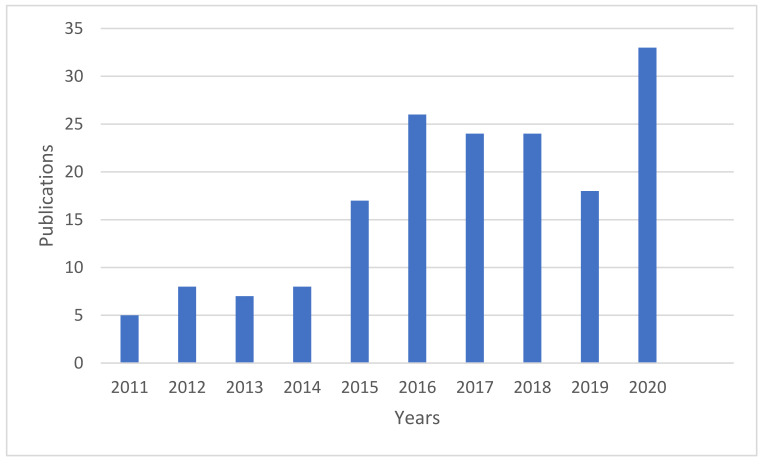
Distribution of annual publications on predicting infectious disease outbreaks research from 2011 to 2020.

Figure 3 below shows the number of publications’ evolutionary trajectories in predicting infectious disease from the year 2011 to 2020 in Africa. It can be observed that the prediction of infectious diseases in Africa is a topic that has not been intensively researched. This is demonstrated by 169 papers and 247 papers published within the past ten years and the past three decades, respectively. In contrast, a staggering 1880 publications on the same topic globally were published in the same period as demonstrated by the retrieval on 1 May 2020 [32]. It follows, therefore, that Africa contributes less than 14% of the publications in this field. Despite this low number for Africa, there has been a constant growth in these publications for the past ten years, except for 2013, 2017, and 2019. The track record of publications on the prediction of infectious disease decreased by 1%, 0.41%, and 0.81% in 2013, 2017, and 2019, respectively.

On the other hand, there has been a significant increase in publications from 2015 to 2018 and 2020. In 2015 there were a total of 17 published documents representing a more than 100% increase from 2014, whereas in 2020 33 documents, which represent an increase of 83.3% from 2019, were published. The year with the highest and lowest publications are 2020 and 2011, respectively. Overall, there is a slow but notable growth of research on the prediction of infectious diseases in Africa—this is demonstrated by the annual average growth rate of published papers of approximately 24.35%.

There is an indication that the increase in the prediction of infectious diseases is associated with the outbreaks of infectious diseases. This is illustrated in Table 1 below, representing the trends of severe outbreaks or first-time detections of infectious diseases in Africa. For instance, as mentioned earlier, there was a significant increase in 2015, 2018, and 2020. It was during this period that certain regions in Africa experienced the largest outbreaks of infectious diseases. For instance, in 2015–2016, Angola and the Democratic Republic of Congo (DRC) experienced a large outbreak of yellow fever, and a devastating epidemic of monkeypox was reported in Nigeria [7].

Moreover, in 2013–2016 the world’s largest Ebola outbreak was reported in several African countries, such as Sierra Leone [41]. In 2017, the worst plague outbreak of the 21st century was reported in Madagascar [42]. There was also an increased interest in researching the prediction of infectious diseases in Africa in 2020 (indicated by a high number of publications this year). It was the same time Africa and the world at large were experiencing the outbreak of COVID-19. However, few 2020 papers are addressing COVID-19 in Africa. The other factor that boosts the publications in the prediction of infectious diseases in Africa is the re-emerging of infectious diseases [32].

**Figure 4:**
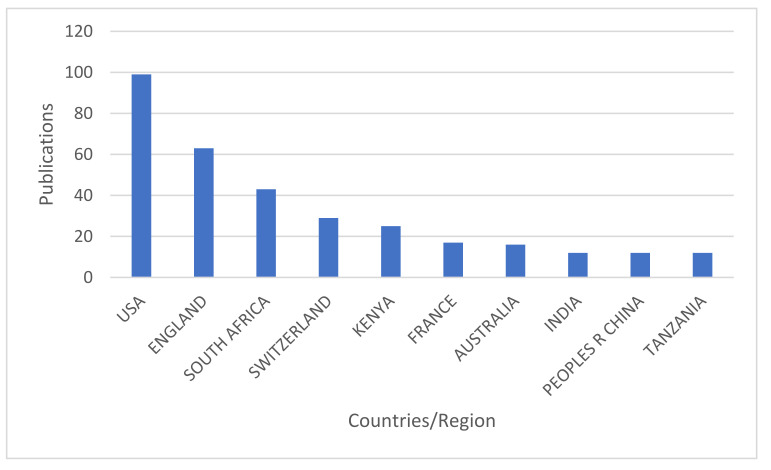
Countries in the top ten ranks in publications on predicting infectious disease outbreaks from 2011 to 2020.

Different countries have contributed to the publications on predicting infectious diseases in Africa. Based on the first author’s country affiliation, the countries were given ranks, and only countries ranking in the top ten were considered and are illustrated in Figure 4 below. The top leading countries are the USA, with 40.1% publications. The second, third, and fourth are England, South Africa, and Switzerland, with 25.5%, 17.4%, and 11.7%, respectively. Even though the USA has the highest number of publications, most countries in the top ten are from Europe and Africa. South Africa, Kenya, and Tanzania are the most productive countries in Africa.

### 3.2. Network Analysis

In the VOSviewer network visualizations, the items (the subject of interest) being analyzed are represented using circles and the lines interconnecting the items indicate the relatedness of the items. The bigger the circle, the higher the item’s weight, indicating the importance or prominence of such an item in the research field [37]. On the other hand, the thicker the line, the higher the association strength exists between the items connected by the line [47].

**Figure 5:**
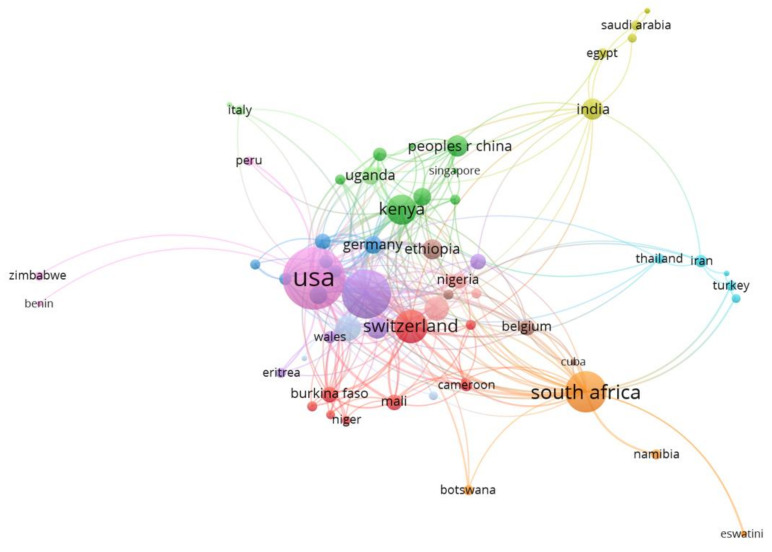
Country collaboration network.

Figure 5 below illustrates the countries’ collaboration networks. The network is presented in terms of association strength of collaborations between the countries. The countries in the same cluster have higher collaboration strength. The network shows 12 clusters. Most of the countries shown in this network also appear in Figure 4 as the top leading countries. These countries are therefore denoted with bigger circles to indicate dominance in the publications. These countries are also very collaborative. The USA (appearing in a light pink cluster) is the most productive and collaborative country followed by England and South Africa.

Although the USA is clustered together with countries such as Benin, Peru, and Zimbabwe, it also shows very strong collaboration with almost every country shown on the map, such as England, Kenya, South Africa, and Switzerland. On the other hand, England (shown in a purple cluster) is clustered with Spain, Tanzania, Wales, and Eritrea. England also shows strong collaboration with the USA and the following African countries: South Africa, Kenya, and Nigeria. The other countries that also show strong collaboration include Kenya, South Africa, Switzerland, Australia, the People’s Republic of China, Tanzania, and France. South Africa and Kenya (appearing in orange and green clusters, respectively) are the most collaborative African countries, followed by Tanzania and Ghana. Furthermore, there is a strong collaboration between Kenya and South Africa. The least collaborative countries in Africa include Namibia, Mozambique, Zimbabwe, Benin, and Botswana, and they mostly collaborate with the USA and/or South Africa.

**Figure 6:**
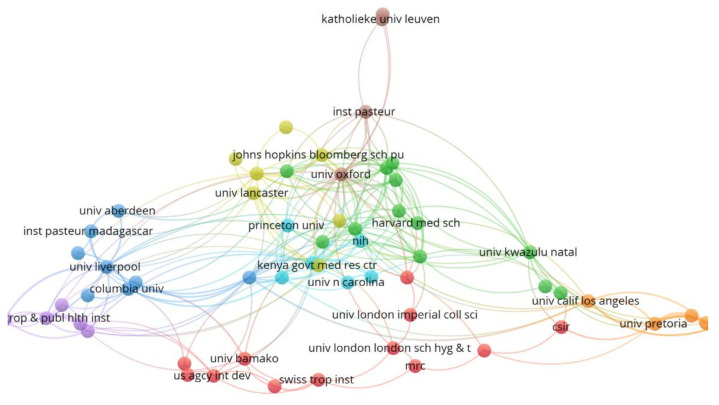
Institution collaboration network.

The results from VOSviewer, which show the visualization of institutions’ collaboration networks, are shown in Figure 6 below. Only institutions meeting the threshold of three published papers were considered. Consequently, 65 of 509 institutions were included in the collaboration network analysis. The institutions were grouped into eight clusters. The cluster with the highest number of collaborative institutions is the red cluster with twelve institutions. Institutions with high strength of collaboration are given the same color and clustered together. For instance, it can be observed that there is a strong collaboration among institutions such as the University of Pretoria, South Africa Weather Service, University of Western Cape, Southern African Science Service Centre, and the University of California, Los Angeles—all appearing in the orange cluster. This cluster mainly depicts the collaboration among South African institutions. On the other hand, the green cluster shows collaboration mainly among the American and British institutions, such as the University of Florida, Harvard University, University of Washington, and the London School of Hygiene and Tropical Medicine.

The top five most collaborative institutions in the prediction of infectious disease in Africa include the University of Oxford, Ministry of Health, Imperial College London, University of Liverpool, Centre for Disease Control and Prevention, Kenya Government Medical Research Centre, University of Southampton, and the University of Pretoria, which collaborate with 43, 25, 25, 21, 21, 20, 20, and 19 institutions, respectively. These institutions are from the USA, England, Kenya, and South Africa, which are among the most productive and collaborative countries, as shown in Figure 4 and Figure 5. Table 2 below shows the total number of documents published by each institution in the top five most productive institutions. It can be noted that most of the productive institutions are also very collaborative, with few exceptions, such as Columbia University, which is very productive but relatively not very collaborative. The most collaborative institutions in Africa include Kenya Government Medical Research Centre, the University of Pretoria, and the University of KwaZulu Natal. These institutions have a strong collaboration with both African institutions and the institutions abroad. The least collaborative institutions include Addis Ababa University, Chinese Academic of Science, and Kwame Nkrumah University of Science and Technology. They are also less productive. It can also be observed that most of the institutions in Figure 6 are from the USA, which implies that there are many scientific research institutions in the USA focusing on predicting infectious diseases in Africa. This is not surprising because Figure 5 above has already highlighted the USA as the biggest contributor to predicting infectious diseases in Africa.

**Figure 7:**
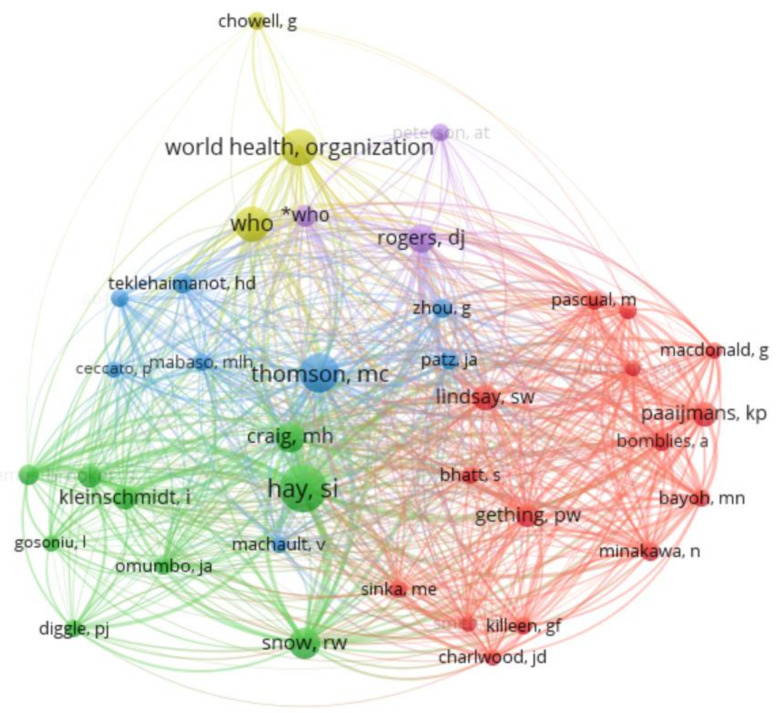
Authors’ co-citation network.

In a co-citation analysis of authors, the authors’ relatedness is determined by the number of times the authors are cited together [39]. Authors’ co-citation determines the knowledge structure, different subfields in specific research, and identifies the most influential or contributing researchers and their interrelationships [48,49]. Figure 7 below illustrates the author co-citation network with five clusters. This network was generated using VOSviewer. Only authors with a minimum of 20 citations were considered, and, consequently, 38 out of 5270 authors were included in this analysis. The authors appearing in the same clusters are mostly cited together in a published paper. It can be observed that the cluster with the highest number of authors is the red cluster, and it contains authors such as Lindsay, S.W., Gething, P.W., and Pascual, M. On the other hand, the purple cluster has the least number of authors, and this includes authors such as the World Health Organization (WHO), Rogers, D.J and Peterson, A.T. It can also be noted that the most contributing authors include Hay, S.I, WHO, Thomson, M.C, Graig, M.H. and Snow, R.W.

### 3.3. Keyword Analysis

**Figure 8:**
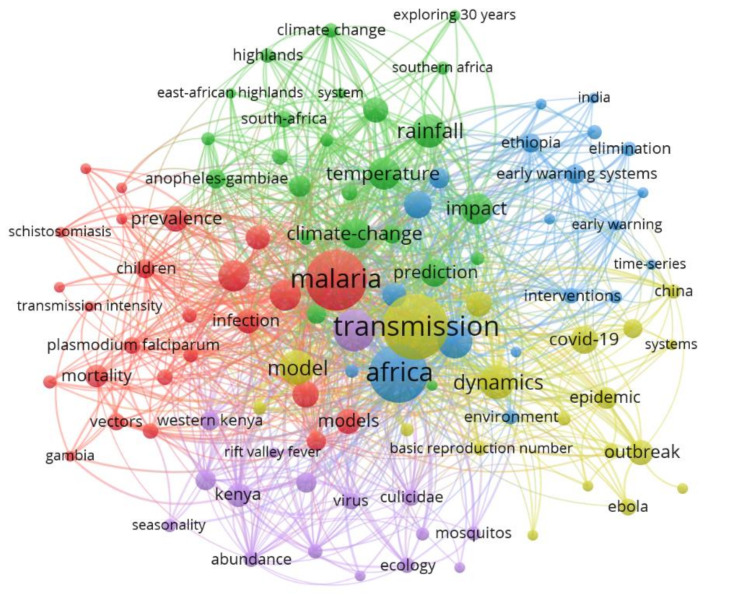
Keywords co-occurrence network.

Keywords co-occurrence counts the number of times two words or more occur together in the same published paper [47]. Keywords co-occurrence analysis can be used to identify the publication trends or knowledge evolution in a specific research field [50]. In this paper, keywords that occurred four times or more in the WOS core database were enrolled in the final analysis and 104 out of 1110 met the threshold. As indicated in Figure 8 below, the keywords in the co-occurrence network are grouped into five clusters. The same cluster keywords are either keywords that strongly relate to each other or appear together frequently in published papers to predict infectious diseases in Africa. The clusters shown in Figure 8 include the red cluster, which has the most elements; therefore, it means that the research in predicting infectious diseases in Africa has ample papers with the keywords in this cluster. The dominant keywords appearing in the red cluster include malaria, mortality, regression, model, disease, schistosomiasis, disease mapping, Sub-Saharan Africa (SSA), and variable selection. This is followed by the green cluster, which contains keywords such as climate change, temperature, El Nino, rainfall, rainfall, and prediction. While the blue cluster is dominated by keywords such as Africa, early warning system, interventions, and patterns, the yellow cluster is dominated by keywords such as transmission, outbreak, Ebola, COVID-19, China, model, and epidemic. On the other hand, the purple cluster covers keywords like Kenya, West Africa, risk, virus, seasonality, and mosquito.

Prominent keywords, represented with bigger circles, reflect research direction and active hot topics in researching infectious disease prediction in Africa. These include Africa, malaria, transmission, climate change, model, COVID-19, epidemic, outbreak, impact, and prediction. These keywords appear in different clusters. This suggests that these keywords have gained widespread attention from researchers. On the other hand, keywords with lower frequency, represented with small circles, are the least researched keywords or are emerging/declining topics. These include disease mapping, regression, variable selection, association, and Zika Virus.

To further understand Figure 8 above, the top 20 keywords appearing in predicting infectious diseases in Africa as per the WoS database are illustrated in Table 3 below. Transmission and malaria are the keywords appearing the most. Furthermore, most keywords are meteorological terms, suggesting that the prediction of infectious diseases in Africa is weather-based. It also emerges that most of the research in this field has been done in Sub-Saharan Africa and Kenya. There are also keywords such as children, which could be because children are usually most affected by infectious diseases in Africa [51]. For example, the WHO 2020 malaria report indicates that about two-thirds of Africa’s malaria-related deaths are among children. The situation was alarming in Sub-Saharan Africa particularly [51]. This could, therefore, explain why there is more research done in Sub-Saharan Africa. Furthermore, it can be seen in Table 3 that climate change has also been considered in the prediction, and there are also very interesting keywords such as model.

### 3.4. Thematic Evolution Analysis

**Figure 9:**
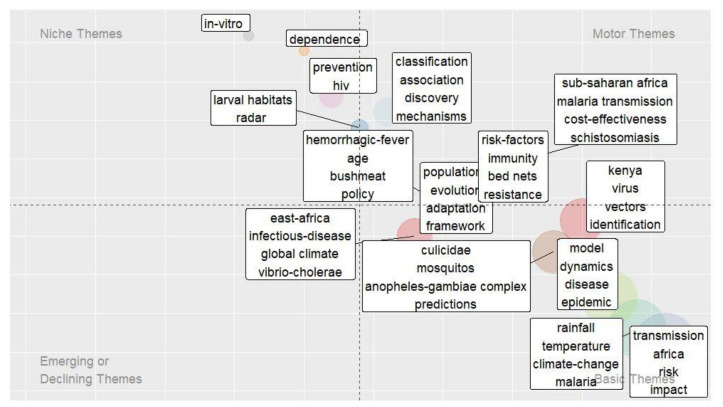
Thematic map of emerging themes in the prediction of infectious diseases research in Africa.

R package was used to perform thematic evolution analysis. Figure 9 below illustrates a thematic map showing the key research themes of the published papers on infectious disease prediction in Africa. A four themes quadrant is provided based on the centrality and density along the x-axis and y-axis, respectively [52]. The circles’ dimensions indicate the number of the published documents corresponding to each keyword, i.e., the bigger the circle, the higher the number of publications equivalent to a certain keyword in each quadrant [53]. The motor themes have strong centrality and high density on the upper right quadrant. These are the themes that are considered well developed and important for the structuring of a research field. These themes can be seen as hot spots in a specific research field. In the research of predicting infectious diseases in Africa, the hot topics include association, classification, discovery, malaria transmission, Sub-Saharan Africa, policy, COVID-19, and risk factors. The themes appearing in the lower right quadrant are called basic and transversal themes and are considered as important but emerging themes that are not well developed. These include keywords such as prediction, rainfall, temperature, climate change, model, infectious diseases, and virus. It can be observed that most of the basic topics about infectious disease prediction in Africa are not well developed; this is indicated, for example, by most of the keywords with the highest frequency (as shown in Figure 5 above) appearing in the lower right quadrant. On the other hand, themes appearing in the lower left quadrant are considered weakly developed themes and have less importance. They have low density and centrality, and this indicates that the themes are either emerging or disappearing topics. As indicated in Figure 9, this quadrant is empty for the prediction of infectious diseases in Africa. The upper left quadrant contains niche themes. These are the themes with high density but low centrality and even though they are well developed they are of marginal importance. These include the following topics: HIV, dependence, prevention, and larval habitats. Generally, the four-theme quadrant shows studies carried in Africa focusing on the use of associations and classification models, and climate change to predict infectious diseases, such as schistosomiasis, but with more focus on malaria.

### 3.5. Number of Received Citations of the Documents and Bibliometric Indices

**Figure 10:**
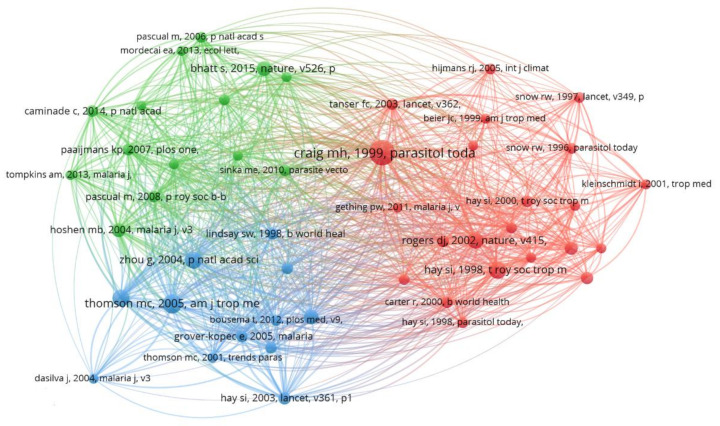
References of co-citation in the prediction of infectious diseases research in Africa.

To generate the reference co-citation network for the prediction of infectious diseases in Africa, the VOSviewer tool was used. In a reference co-citation analysis, the items relatedness is based on the number of times the papers are cited together [32]. The idea behind the reference co-citation analysis is to find groups of similar papers or papers that address the same issues because the more two papers are co-cited the more similar they are [54]. In this research study, out of 7594 cited references, 50 papers met the threshold of over ten co-citations. It can be observed in Figure 10 below that the most influential paper in the research of predicting infectious diseases is by Craig M.J. 1999 (A climate-based distribution model of malaria transmission in sub-Saharan Africa) as per the data collected from WoS. Craig M.J. 1999 is the most cited and co-cited paper; this is indicated by a bigger circle representing this paper and the large number of links from this node to other nodes in the network. For readability purposes, the information in Figure 10 was illustrated in Table 4 below, and the top ten most cited papers are shown. From Table 4 below, it can be noted that most of the papers with a significant contribution are journal papers; there is only one conference paper. It can also be noted that these papers deal with the prediction of malaria. This confirms the findings in [55], which indicates that malaria is one of the infectious diseases highly researched in Africa. It can also be seen that almost all the papers in Table 4 are co-authored. Additionally, it can be observed in these highly cited papers that African researchers are highly underrepresented as authors and first authors. It is also interesting to note that the number of citations of these highly cited papers from VOSviewer differs significantly from the citations given by Google Scholar.

## 4. Discussion

### 4.1. Outbreaks-Driven Research

Table 1 reveals some of the largest outbreaks of infectious diseases and their resulting impact on Africa’s continent from 2010 to 2021. This review indicates that the frequent and severe outbreaks of infectious diseases in Africa have increased the research and publications in predicting infectious diseases in Africa. These findings synchronize with the bibliometric study carried out by [33], which showed a remarkable upsurge in the publications of papers in the research on Ebola from 2013 to 2015. The papers in Scopus and WoS increased from 54 and 52 to 897 and 953, respectively, within this period (2013 to 2015). As mentioned earlier, in 2013, Africa was hit by the worst outbreak of Ebola in history. This indicates that disease outbreaks trigger research interests. Most research interests revolve around the prediction of outbreaks and search for ways to minimize and control the transmission of such infectious diseases.

### 4.2. Under-Representation of African Authors

Even though Africa has the highest infectious diseases burden, it contributes very little to research on predicting infectious diseases in Africa. African researchers are highly underrepresented as authors and as the first or last authors in this research field. These findings coincide with the findings from several studies that highlight the continuous low contribution of Africa in the research. Naidoo et al. [65] conducted a bibliometric review of authorships for infectious disease research done in Africa from 1980 to 2016 and published in the top ten medical journals.

This study reveals that Africa’s research output contributes less than 1% to the world’s health research and 3.9% to the articles on COVID-19 in Africa. On the other hand, similar results were observed in the study conducted by [66] on the presentation of African authors in high-impact geoscience literature. Only 30% of the high impact geoscience articles on African topics had an African author and these articles contribute only 3.9% of the 3573 articles that are published every year globally.

It is interesting to note that most of the research done in Africa is the collaborative work between African and Western authors, as depicted in Figure 6 above and also as highlighted by the literature [55]. Although this collaboration is a positive initiative to increase the research capacity in Africa, the inequality in the partnership poses the threat of power imbalance. This can lead to conducting inappropriate research projects that ignore the local research needs or lack direct benefits to the locals in Africa [65]. Opinion, contextual guidelines, and commentary play a vital role in health policy and it is a concern to note that 90% of the opinion-based articles on COVID-19 in Africa are by non-African authors [65]. Western nations fund most research projects in Africa and often dictate the research agenda, explaining the inequality in the research partnership in research done in Africa [33,55]. When Africa is underrepresented in research, it has little or no say in providing advice on system response, clinical care, and policy making regarding infectious disease outbreaks in Africa or globally.

The African authors are underrepresented in research for various reasons. There is a brain drain of scientists from Africa and low investments in research by Africa [66,67,68]. African researchers have high teaching loads and receive little incentives, which negatively impacts African research output [68].

On the other hand, the network analysis results reveal that the USA and European countries dominate research in this field. The most productive and highly cited authors and the most collaborative and productive institutions are also from Western nations. These results correspond with the findings of [32,33] that indicate that the USA and European countries dominate the research on predicting infectious diseases and that they are the global scientific leaders in other research fields too. This can be attributed to the level of their research effort, funds, ref. [69], and the devastating impact of infectious diseases and their possibility of spreading globally, which calls for an international intervention [55].

Nevertheless, South Africa and Kenya show significant contributions and high collaboration with other countries. It is not surprising to see South Africa as the most productive and collaborative African country in this research field because South Africa accounts for one-third of Africa’s publications [55]. Additionally, all the highly cited papers are collaborative work. Predicting infectious disease can be viewed as transdisciplinary; therefore, it requires knowledge from multiple disciplines such as infectious disease, microbiology and science, and technology [32].

### 4.3. Domination of Malaria in the Research Theme

According to the hot topics identified from keywords and thematic evolution analysis (Table 3 and Figure 8 and Figure 9), malaria is the most frequent keyword and a focus area of the highly cited documents. This is not surprising because 90% of malaria incidences are in Africa [70]. Moreover, the researchers have also focused on the transmission and impact of malaria over other infectious diseases. These findings synchronize with the discovery by [38] that reveal malaria as the most encountered infectious disease in the infection-related literature. In addition, factors such as climate change, cost-effectiveness, and rainfall have been considered in predicting malaria. This is because there is ample evidence linking malaria incidence to climate variations like temperature, rainfall, and humidity [15,38]. The over-focus on malaria over other infectious diseases may introduce bias in the results. The immense focus on malaria in the research of infectious disease prediction could also be attributed to the high funding for research in malaria [55]. It can be noted that COVID-19 appears in the top 20 keywords in the research of predicting infectious diseases in Africa. Even though the frequency of the COVID-19 keyword is relatively low compared to malaria, it is interesting to see that Africa is making efforts to combat COVID-19. However, as highlighted in 4.2, Africa contributes too little to combat infectious diseases such as COVID-19.

### 4.4. Slow Adoption of Fourth Industrial Revolution Technologies

Fourth Industrial Revolution technologies such as artificial intelligence, machine learning, big data, remote sensing, wireless sensor networks, and mobile technologies have been widely used to develop early warning systems globally [22,62,71,72]. However, these tools are underutilized in predicting infectious diseases in Africa. Even though some of these 4IR tools, such as machine learning and artificial intelligence keywords, were used as search strings to extract papers from WoS, this search did not yield significant results. Only keywords with a minimum of four occurrences were considered in the keyword analysis, and none of the 4IR tools were represented. Even though researchers such as [15,71,72] used machine learning to predict malaria, the minimal presence of the keywords ‘machine learning’ in the search results indicates that machine learning in the prediction of infectious diseases in Africa remains modest. With the growth in big data, accurate medical and climate data analysis allows predicting and early detection of infectious diseases associated with climate change [73]. On the other hand, mobile technology and artificial intelligence have been considered effective tools to tackle complex problems such as predicting infectious disease outbreaks [74].

### 4.5. Recommendations: Key Emerging Themes and the Way Forward

According to the results in this review, the researchers in the field of predicting infectious diseases in Africa have focused on classification, associations, COVID-19, cost-effectiveness, schistosomiasis, malaria transmission, policy, and risk factors. These topics are hotspots in researching the prediction of infectious diseases in Africa, and they are important for the structuring of this research field. However, researchers are also shifting their focus to new interesting subtopics to predict infectious diseases in Africa. These include weather-based prediction, rainfall, model, temperature, climate change, malaria, virus, prevalence, intensity, and epidemic. These keywords are considered important and emerging but not well-developed themes in this research field. This implies that researchers in this field focus primarily on weather-based prediction of infectious diseases, malaria, as mentioned earlier, and incorporated mainly meteorological factors such as rain and temperature.

Based on the results and the discussions articulated in the previous sub-sections, this review provides the following recommendations in predicting infectious diseases in Africa:

- There is a dire need to promote publications and equitable partnership between African researchers and other researchers in predicting African’s infectious diseases. Ways to achieve this include but are not limited to: (1) collaboration from expertise in different fields since predicting infectious diseases is interdisciplinary as mentioned earlier; (2) more funding from African agencies to promote equitable research collaboration [55] emphasize that mutually beneficial partnerships between African countries and Western nations can improve research capacity and address global health challenges.
- Future studies should focus more on predicting infectious diseases such as COVID-19 and Ebola, which, unlike malaria, have not received enough attention from researchers regardless of their continuous devasting impacts [75]. The ongoing outbreak and the devastating impacts of COVID-19 have indicated that early warning systems are now needed more than ever to build preparedness for infectious diseases.
- Considering that researchers in predicting Africa’s infectious diseases have focused on weather-based prediction systems considering mainly meteorological factors, future studies must incorporate other factors ranging from hydrological, environmental, international travel, human demographics and behavior, social media, and lack of political will [7]. Other researchers outside Africa have already considered these factors and have enhanced the precisions of the prediction models [11,28]. Since factors that influence infectious diseases vary from one country/region to another [72], future studies should also investigate which factors are important for predicting infectious diseases in a specific region/country in Africa.
- There is also a need to explore further machine learning, artificial intelligence and other 4IR tools to find which technological tools can efficiently and effectively monitor and predict infectious diseases in Africa. These technological tools can be pivotal to reinforce the capacity of traditional surveillance systems [26].

This review, however, has some limitations. It has used data from WoS only, resulting in less perfect bibliometric analysis results. In future studies, we will incorporate databases such as Scopus for a perfect comprehensive review. Nevertheless, this review used two rigorous bibliometric software; VOSviewer and R studio and provided the bird’s-eye view and understanding of the available knowledge in researching the prediction of infectious diseases in Africa.

## 5. Conclusions

With the focus on the papers predicting infectious diseases in Africa, this review conducted a knowledge mapping analysis of literature to reveal the current research status and research hotspots and gaps in this field in Africa. This paper concludes that: (1) the severe outbreaks of infectious diseases in Africa have increased scientific publications during the past decade; (2) malaria prediction dominates the research theme; (3) the identified relevant hotspots include malaria, models, classification, associations, COVID-19, and cost-effectiveness; (4) like many other advancements, Africa has not kept pace in the research of predicting infectious diseases in terms of research output and the use of 4IR tools; (5) Africa has focused on weather-based prediction systems considering mainly meteorological factors. These findings call to African researchers, institutions, and funding agencies to invest more in this research field. To African researchers, there is a need to explore 4IR tools and consider integrated approaches for predicting infectious diseases.

This study has determined the nature of predicting infectious disease research in Africa, the paradigmatic shift, current challenges, and research opportunities, hence providing valuable information to African researchers, institutions, and funding agencies. This paper can also be a useful starting point for researchers interested in predicting infectious diseases in Africa. Moreover, it contributes towards building empirical evidence on predicting infectious diseases in the domain of Africa. These contributions support the efforts combating infectious diseases, especially in the African region, which continues to bear the burden of infectious diseases.

## Figures and Tables

**Table 1:** Severe outbreaks/first outbreak detection of infectious diseases in Africa from 2010 to 2020.

Infectious Diseases	Period of Occurrence	Countries/Regions	Impact	Sources
Plague	2017	Madagascar	2348 confirmed and 202 deaths	[42]
Measles	2010–2013	DRC	Largest outbreak: 294,455 cases, 5045 deaths	[43]
Yellow Fever Virus	2015–2016	Angola, DRC	Largest outbreak: 7334 suspected cases, 393 deaths	[44]
Ebola	2013–2016	Guinea, Sierra Leone, Liberia	Largest outbreak: 28,646 cases and 11,323 deaths	[7]
Monkeypox	2017	Nigeria	Largest outbreak: 146 suspected cases and 42 confirmed cases, 1 death	[45]
Zika Virus	2015–2016	Cabo Verde	First outbreak detection in Africa, 7580 Zika virus suspected cases	[46]
COVID-19	2019–4 January 2022 (ongoing)	Africa	7,164,485 confirmed cases and 155,675 deaths	[6]

**Table 2:** Top four most collaborative institutions in the research of predicting infectious diseases in Africa.

Rank	Institution	Published Papers	Citations	Total Link Strength
1	University of Oxford	20	1309	43
2	Columbia University	15	654	17
3	University of Liverpool	14	769	21
4	University of Pretoria	12	73	19
5	Kenya Government Medical Research Centre	11	676	20
5	Ministry of Health	11	614	25

**Table 3:** Top 20 keywords in the research of predicting infectious diseases in Africa.

Rank	Keyword	Occurrences	Total Link Strength	Rank	Keyword	Occurrences	Total Link Strength
1	transmission	68	346	11	Plasmodium falciparum	20	111
2	malaria	60	334	12	epidemiology	20	103
3	Africa	57	304	13	climate change	19	100
4	risk	25	191	14	patterns	17	110
5	model	25	129	15	prediction	17	87
6	climate	24	137	16	COVID-19	17	33
7	dynamics	24	122	17	disease	16	63
8	rainfall	23	133	18	variability	15	87
9	temperature	22	147	19	outbreak	15	57
10	impact	20	113	20	epidemic	14	89

**Table 4:** The top-cited papers in the field of prediction of infectious diseases in Africa.

Rank	Authors and Year	Paper Title	Paper Type	Citations from VOSviewer	Citations from Google Scholar
1	Craig, M.H., Snow, R.W. and le Sueur, D., 1999. [56]	A climate-based distribution model of malaria transmission in sub-Saharan Africa.	Journal: Parasitology today	45	1036
2	Thomson, M.C., Mason, S.J., Phindela, T. and Connor, S.J., 2005. [57]	Use of rainfall and sea surface temperature monitoring for malaria early warning in Botswana.	The American Journal of Tropical Medicine and Hygiene	29	279
3	Thomson, M.C., Doblas-Reyes, F.J., Mason, S.J., Hagedorn, R., Connor, S.J., Phindela, T., Morse, A.P. and Palmer, T.N., 2006. [58]	Malaria early warnings based on seasonal climate forecasts from multi-model ensembles.	Nature	24	5
4	Zhou, G., Minakawa, N., Githeko, A.K. and Yan, G., 2004. [59]	Association between climate variability and malaria epidemics in the East African highlands.	Conference paper: Proceedings of the National Academy of Sciences	23	543
5	Hay, S.I., Snow, R.W. and Rogers, D.J., 1998. [60]	Predicting malaria seasons in Kenya using multitemporal meteorological satellite sensor data.	Transactions of the Royal Society of Tropical Medicine and Hygiene	23	291
6	Rogers, D.J., Randolph, S.E., Snow, R.W. and Hay, S.I., 2002. [61]	Satellite imagery in the study and forecast of malaria.	Journal: Nature	21	556
7	Teklehaimanot, H.D., Lipsitch, M., Teklehaimanot, A. and Schwartz, J., 2004. [62]	Weather-based prediction of Plasmodium falciparum malaria in epidemic-prone regions of Ethiopia I. Patterns of lagged weather effects reflect biological mechanisms.	Malaria journal	19	241
8	Hoshen, M.B. and Morse, A.P., 2004. [63]	A weather-driven model of malaria transmission.	Malaria journal	17	321
9	Kleinschmidt, I., Bagayoko, M., Clarke, G.P.Y., Craig, M. and Le Sueur, D., 2000. [64]	A spatial statistical approach to malaria mapping.	International Journal of Epidemiology	16	10
10	Hay, S.I., Were, E.C., Renshaw, M., Noor, A.M., Ochola, S.A., Olusanmi, I., Alipui, N. and Snow, R.W., 2003. Forecasting, warning, and detection of malaria epidemics: A case study. The Lancet, 361(9370), pp. 1705–1706.	Forecasting, warning, and detection of malaria epidemics: A case study.	The Lancet journal	14	134

## Data Availability

Data is contained in the supplementary material.

## Supplementary Materials

The following supporting information can be downloaded at https://www.mdpi.com/article/10.3390/ijerph19031893/s1.
